# Improvement of the Effectiveness of HER2+ Cancer Therapy by Use of Doxorubicin and Trastuzumab Modified Radioactive Gold Nanoparticles

**DOI:** 10.1021/acs.molpharmaceut.3c00414

**Published:** 2023-08-22

**Authors:** Kinga Żelechowska-Matysiak, Evangelia-Alexandra Salvanou, Penelope Bouziotis, Tadeusz Budlewski, Aleksander Bilewicz, Agnieszka Majkowska-Pilip

**Affiliations:** † Centre of Radiochemistry and Nuclear Chemistry, Institute of Nuclear Chemistry and Technology, 03-195 Warsaw, Poland; ‡ Institute of Nuclear & Radiological Sciences & Technology, Energy & Safety, N.C.S.R. “Demokritos”, Agia Paraskevi, 15341 Athens, Greece; § Isotope Therapy Department, National Medical Institute of the Ministry of the Interior and Administration, 02-507 Warsaw, Poland

**Keywords:** gold-198, gold nanoparticles, doxorubicin, trastuzumab, HER2+ cancer, targeted therapy

## Abstract

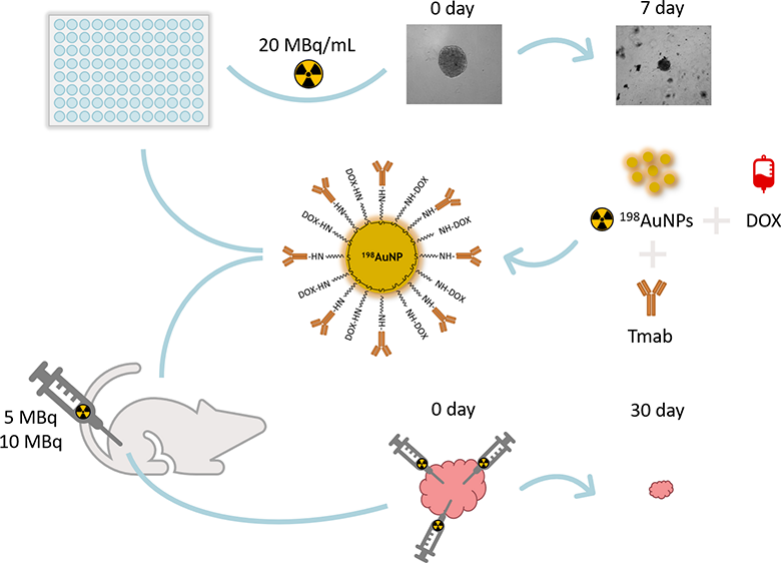

In the present article, we describe a multimodal radiobioconjugate that contains a chemotherapeutic agent (doxorubicin, DOX), a β-emitter (^198^Au), and a guiding vector (trastuzumab, Tmab) for targeted therapy of cancers overexpressing HER2 receptors. To achieve this goal, radioactive gold nanoparticles (^198^AuNPs) with a mean diameter of 30 nm were synthesized and coated with a poly(ethylene glycol) (PEG) linker conjugated to DOX and monoclonal antibody (Tmab) via peptide bond formation. In vitro experiments demonstrated a high affinity of the radiobioconjugate to HER2 receptors and cell internalization. Cytotoxicity experiments performed using the MTS assay showed a significant decrease in the viability of SKOV-3 cells. A synergistic cytotoxic effect due to the simultaneous presence of DOX and ^198^Au was revealed after 48 h of treatment with 2.5 MBq/mL. Flow cytometry analysis indicated that DOX-^198^AuNPs-Tmab mainly induced cell cycle arrest in the G2/M phase and late apoptosis. Dose-dependent additive and synergistic effects of the radiobioconjugate were also shown in spheroid models. Ex vivo biodistribution experiments were performed in SKOV-3 tumor-bearing mice, investigating different distributions of the ^198^AuNPs-DOX and DOX-^198^AuNPs-Tmab after intravenous (i.v.) and intratumoral (i.t.) administration. Finally, in vivo therapeutic efficacy studies on the same animal model demonstrated very promising results, as they showed a significant tumor growth arrest up to 28 days following a single intratumoral injection of 10 MBq. Therefore, the proposed multimodal radiobioconjugate shows great potential for the local treatment of HER2+ cancers.

## 1 Introduction

Cancer remains a major threat to human health worldwide. It is estimated that there will be 22 million new cancer cases by 2035. While modern medicine deals with solid tumors relatively well, in the case of metastatic cancers, therapeutic options are limited, with chemotherapy and immunotherapy among the key therapeutic options. Frequently used chemotherapeutics, such as doxorubicin (DOX), paclitaxel, docetaxel, and cisplatin, are able to kill cancer cells rapidly upon uptake. The extensive use of DOX for over 50 years has substantially improved cancer survival statistics. However, the rapid clearance and nonspecific distribution of these chemotherapeutics and serious side effects associated with these drugs are limiting factors in the application of these treatment regimens. Additionally, prolonged drug use often induces strong resistance pathways in cancer cells against chemotherapeutics. In order to avoid these side effects, suboptimal doses are used, which often result in therapeutic failure and bad prognosis. Therefore, in order to increase the effectiveness of the therapy, two or more methods of treatment are proposed, either simultaneously or sequentially.

The most frequently applied multimodal treatment encompasses the use of chemotherapy and immunotherapy^1^ in a single drug form. As mentioned in several papers, the combination of chemotherapy and immunotherapy may produce synergistic therapeutic effects where 1 + 1 > 2.^2−4^ In 2013, the Food and Drug Administration (FDA) approved the use of the drug ado-trastuzumab emtansine (Kadcyla) to treat HER2-positive breast cancer. Kadcyla is a drug that contains the monoclonal antibody trastuzumab, an immunotherapeutic agent blocking the activity of the HER2 protein on cancer cells, and the chemotherapeutic agent emtansine. Once the antibody binds to the HER2 receptor, emtansine is released into the cells. Other multimodal systems currently under investigation may include gene therapy, chemotherapy, and radionuclide therapy. These treatment options can be combined with magnetic hyperthermia induced via either superparamagnetic iron oxide nanoparticles (SPIONs) or gold nanoparticles (AuNPs) excited by near-infrared (NIR) or microwave radiation.

The enhancement of external radiation or internal radionuclide therapy could be achieved with the simultaneous application of chemotherapeutics. This is a treatment option for patients with aggressive metastatic tumors, where it is impossible to achieve response to a single treatment strategy and complementary/synergistic effects of both therapeutic strategies is observed.^5^ This effect is most commonly mediated by interference with cellular repair processes, which normally repair sublethal DNA damage caused by ionizing radiation. When the cell enters the mitosis phase, the DNA strand breaks (which may be repaired), become fixed and from sublethal damage, the result is eventually lethal damage.^6^ Also, enhancement of the chemotherapeutic effect by ionizing radiation allows for much lower doses of chemotherapeutics. This strategy of treating advanced cancers by external radiation therapy (teleradiotherapy), supported by chemotherapy, is widely used in modern nuclear medicine. Therefore, combining therapeutic radiopharmaceuticals with a selected chemotherapeutic is the next rational step along this path.

Nanotechnology gives combined radionuclide therapy an additional perspective through the assembly of multiple monotherapies on a single nanostructured platform. Carrier structures such as polymers, liposomes, cubosomes, and various organic and inorganic nanoparticles allow the encapsulation of a wide spectrum of chemotherapeutics, which can be further labeled with radionuclides and targeting molecules, leading to complementary therapeutic effect.^7,8^ Few publications describe in vitro and in vivo studies of liposomes that contain both chemotherapeutics and β^–^ radiation emitters. Studies of Gao et al.^9^ indicate that combination therapy of ^131^iodine-labeled nanoliposomes loaded with doxorubicin (^131^I-DOX-NL) resulted in higher survival rates in U87 tumor models, and led to shrinking of the tumors, when compared to the application of monotherapy (^131^I-NL or DOX-NL). Chen et al. described rhenium-188 (^188^Re) and doxorubicin encapsulated liposomes for therapy of colorectal adenocarcinoma (HT-29 cells).^10^ Although this system had no targeting molecules, it exhibited a high tumor/background ratio. In another publication, a folate-functionalized lipid nanoparticle comprised of doxorubicin and yttrium-90 (^90^Y) for the targeted therapy of carcinoma CNE1 animal model was developed.^11^ Tumor growth was significantly suppressed in comparison with the control groups. Furthermore, Zolata et al.^12^ successfully performed targeted drug delivery along with hyperthermia, radioimmunotherapy and controlled chemotherapy of tumors using ^111^In-labeled multifunctional SPIONs functionalized with doxorubicin and trastuzumab.

Radioactive gold nanoparticles have attractive prospects for cancer therapy, since gold-198 (^198^Au) and gold-199 (^199^Au) have suitable half-lives and emit β^–^ particles of desirable energy (^198^Au: *t*_1/2_ = 2.7 days, β_max_ = 0.96 MeV; ^199^Au: *t*_1/2_ = 3.14 days, β_max_ = 0.46 MeV) in addition to γ-ray photon emission for single photon emission computed tomography (SPECT). In particular, ^198^Au can be easily obtained at very high activities by thermal neutron irradiation of monoisotopic target gold-197 (^197^Au). The high cross-section for the ^197^Au(*n*,γ)^198^Au nuclear reaction (98.7 barn) allows the production of 350 GBq of ^198^Au in a high neutron flux reactor (1 mg Au target, 1.5 × 10^15^ n/cm^2^ /s neutron flux, 70 h irradiation). After irradiation under these conditions, ∼5% of the gold atoms are radioactive. The use of such irradiated gold material for AuNPs synthesis allows the formation of 5 nm AuNPs containing 200 atoms of radioactive ^198^Au and, in the case of 20-nm-sized AuNPs, one nanoparticle will contain 5000 ^198^Au nuclides. Radioactive AuNPs can accumulate in the tumor via the enhanced permeability and retention (EPR) effect; however, tumor retention can be significantly increased by the conjugation of a suitable targeting vector such as a monoclonal antibody, peptide, or biologically active small molecule.

We herein describe a multimodal agent where three different types of therapy—namely, radiotherapy, chemotherapy, and immunotherapy along with a guiding vector—were incorporated onto a single platform, which is the gold nanoparticle, in order to improve therapeutic efficacy for cancer treatment. More specifically, radiolabeled ^198^Au nanoparticles were synthesized and modified with PEG linked to the chemotherapeutic drug doxorubicin and the monoclonal antibody trastuzumab, which will specifically bind the radiobioconjugate to HER2-positive tumor cells. The synthesized radiobioconjugate has been designed to exhibit therapeutic trimodality: radiotherapeutic, chemotherapeutic, and immunotherapeutic by blocking the activity of the HER2 protein. The nanoconjugate was thoroughly characterized by various analytical techniques, and its suitability for clinical administration was demonstrated.

## 2 Materials and Methods

### 2.1 Materials

Sodium hydroxide (NaOH), trisodium citrate dihydrate (C_6_H_9_Na_3_O_9_), polyethylene glycol (HS-PEG-COOH, 5 kDa), and doxorubicin hydrate were purchased from Millipore Sigma (St. Louis, MO, USA). Alpha-pyridyl-2-disulfid-omega-carboxy succinimidyl ester poly(ethylene glycol) (OPSS-PEG-NHS,5 kDa) was from Creative PEGworks (Chapel Hill, NC, USA), hydrochloric acid (HCl, 35–38%) and nitric acid (V) (HNO_3_, 65%) were from Chempur (Piekary Śla̧skie, Poland). Trastuzumab was isolated from Herceptin (Roche Pharmaceuticals, Basel, Switzerland); *N*-(3-(dimethylamino)propyl)-*N*_0_-ethylcarbo-diimide hydrochloride (EDC, >99%) from Thermo Scientific (Rockford, IL, USA); solid gold-197 target (99.99%). Ultrapure deionized water was obtained using the ultrapure water (18.2 MΩ cm) filtration system Hydrolab (Straszyn, Poland).

SKOV-3 (ovarian cancer, HER2+) and MDA-MB-231 (breast cancer, HER2-) cell lines were cultured according to the American Type Culture Collection protocol (ATCC, Rockville, MD, USA). Cells were cultured in an incubator at 37 °C and a humidified atmosphere of 5% CO_2_, using McCoy’s 5A and Dulbecco’s modified eagle medium (DMEM) enriched with penicillin and streptomycin (100 IU/mL) and 10% of heat-inactivated fetal bovine serum. For trypsinization, trypsin EDTA solution C (0.25%) was used. These reagents were obtained from Biological Industries (Beth Haemek, Israel). For in vitro and in vivo studies, the following reagents were used: phosphate-buffered saline (PBS), from Biological Industries (Beth Haemek, Israel); CellTiter96 Aqueous One Solution Reagent (MTS compound), from Promega (Mannheim, Germany); FITC Annexin V, propidium iodide staining solution (PI), and 10× Annexin V Binding Buffer, from BD Biosciences (Becton, Dickinson and Company, Franklin Lakes, NJ, USA), ethanol (ChemPur, Piekary Śla̧skie, Poland); saline solution (NaCl; Polatom, Otwock-Świerk, Poland).

### 2.2 Production of ^198^Au

Gold-198 was obtained by hourly neutron irradiation of a solid gold-197 target at the MARIA Research Reactor in the NCBJ (National Centre for Nuclear Research) in Otwock-Świerk, Poland. Approximately 5.65 GBq (after 12 h of cooling) was obtained from 20 mg of gold target. The radioactive gold target was dissolved in 200–400 μL of aqua regia (HNO_3_:HCl = 1:3) and heated at 120 °C until evaporation. To remove the remaining nitrates, HCl (0.05 M) was added (3 × 200 μL), and the sample was evaporated after each addition of HCl. The procedure was then repeated using water, and, finally, the solution was left in the water. The following steps including synthesis, characterization of the size, zeta potential, sphericity of the nanoparticles, as well as verification of the amount of conjugated doxorubicin to AuNPs and quantitative analysis of conjugated trastuzumab particles to AuNPs have been previously described.^13^

### 2.3 Binding Studies

Twenty four hours (24 h) before the experiment, SKOV-3 and MDA-MB-231 cells (6 × 10^5^ cells) were seeded into 6-well plates (TPP, Switzerland) and stored in an incubator. Prior to the addition of the compound DOX-^198^AuNPs-Tmab, the incubation medium was removed, and cells were washed with PBS. Incubation with different concentrations of the compound was carried out for 1.5 h. The medium was collected and the cells were washed again with PBS. To collect the cell-bound fraction, 1 M NaOH was used. Media and cell activities were measured on a Gamma Wizard^2^ Detector counter (PerkinElmer, Waltham, MA, USA). To determine specific binding, the ratio between total and nonspecific binding was calculated. For the blocking experiment, a 100 molar excess of unconjugated trastuzumab was used to block HER2 receptors.

### 2.4 Internalization Studies

Cells for the internalization assay were prepared in the same way as cells for the binding assay. After the medium was removed, 5 nM DOX-^198^AuNPs-Tmab was added and incubated for 1 h at 4 °C. The fraction was then collected, the cells were rinsed with PBS and fresh medium was added. After the designated time points (1, 6, 18, and 24 h), fractions were collected, cells were washed with PBS and 0.05 M glycine (pH 2.8) was added twice. Cells with glycine were kept in the refrigerator for 5 min. After collecting another fraction, 1 M NaOH was added and the cells were harvested. A 100-molar excess of free trastuzumab was used to check nonspecific binding. Samples were measured using a Gamma Wizard^2^ detector counter.

### 2.5 Cytotoxicity Studies

For the cytotoxicity assay, SKOV-3 (2.5 × 10^3^) and MDA-MB-231 (2 × 10^3^) cells were seeded into 96-well plates. After 24 h, compounds were added and incubated for 24, 48, and 72 h. Prior to adding 20 μL of MTS reagent (CellTiter96 Aqueous Nonradioactive Cell Proliferation Assay), cells were washed with PBS and a new medium was added. Absorbance was measured at 490 nm.

### 2.6 Flow Cytometry: Apoptosis and Cell Cycle Assay

For apoptosis and cell cycle analysis, SKOV-3 cells were prepared following the same procedure as that described for the binding assay. Analysis was performed using flow cytometry after 24 h. Cells were treated with trypsin and then with cold PBS and 1X Annexin V Binding Buffer. Finally, 5 μL of fluorescein isothiocyanate (FITC) with 5 μL of propidium iodide (PI) was added, and the cells were kept for 15 min in an incubator. Cells for cell cycle analysis were prepared in the same way, but after using cold PBS, cells were resuspended in 70% cold ethanol and kept in the freezer for 1.5–2 h. Before analysis, ethanol was removed, cells were rinsed with PBS and 20 μL of PI with 2 μL of RNase were added. Samples were analyzed on a FACSCelesta instrument (BD Biosciences, San Jose, CA, USA), together with FACSDiva v8.0 software (BD Biosciences, San Jose, CA, USA).

### 2.7 Spheroids

SKOV-3 cells were cultured for 5 days in a 96-well ultralow adhesion surface plate (Corning, NY, USA). After addition of the compounds, the surface area of the spheroids was measured for 1 week. The medium was replaced with fresh medium every 2 days. Spheroids were analyzed using a Primovert Color Axiocam 305 microscope (Zeiss, Jena, Germany) with ZEN 3.0 lite software (Zeiss, Jena, Germany).

### 2.8 Ex Vivo Biodistribution Studies

Animals used for the biodistribution studies were obtained from the breeding facilities of the Institute of Biosciences and Applications, NCSR “Demokritos”. This experimental animal facility is registered according to the Greek Presidential Decree 56/2013 (Reg. No. EL 25 BIO 022), in accordance with European Directive 2010/63 on the protection of animals used for scientific purposes, which is harmonized with national legislation. The animal testing protocol was approved by the Department of Agriculture and Veterinary Services of the Prefecture of Attiki.

For the study, 4 × 10^6^ SKOV-3 cells were implanted into female SCID mice (8 weeks old) in the right foreleg. After 20 days, when the tumors reached a volume of ∼300 mm^3^, the experiment was initiated. Mice were divided into three groups (*n* = 3 per group). The first group (control) received 100 μL of ^198^AuNPs-DOX intravenously at a dose of 5.75 ± 0.23 MBq. Another group received 100 μL of DOX-^198^AuNPs-Tmab intravenously at a dose of 6.16 ± 0.20 MBq, while the third group received the same compound directly into the tumor (intratumoral administration, i.t. 50 μL/6.30 ± 0.18 MBq per mouse). The activity of the syringe and needle was measured before and after radiotracer administration to calculate the actual dose received. At 4, 24, and 48 h after injection, mice were euthanized by isofluorane inhalation and the following tissues/organs were removed, weighed, and measured for activity in an automatic γ-counter (Cobra II, Canberra, Packard): blood, liver, heart, kidneys, stomach, intestines, spleen, muscle, lungs, bone, pancreas, and tumor. Tail counts of each mouse were also measured. All measurements were corrected for background and radioactive decay. The percentage of injected activity per gram (% IA/g) was calculated and reported with standard deviation, corrected against the residual activity in the tail, using an appropriate standard.

### 2.9 Therapeutic Efficacy Studies

Mice subjected to the therapeutic efficacy study were prepared in the same way as mice for the biodistribution study. After 20 days from the implantation of SKOV-3 cells, tumors reached a volume of ∼300 mm^3^. Mice were randomly divided into three groups (*n* = 3 per group). The first group (Control Group) received an i.t. saline injection, while the other two groups were i.t. treated with 50 μL of DOX-^198^AuNPs-Tmab at doses of 5 MBq (5.16 ± 0.10 MBq) and 10 MBq (9.99 ± 0.22 MBq). Tumor dimensions were measured with caliper for 28 days (measurements were taken every 3–4 days). Data are presented as a Tumor Growth Index (TGI), which was calculated by dividing the tumor volume measured on each day by the tumor volume measured on Day 0 (the day of injection prior to administration).

### 2.10 Statistical Analysis

Statistical analysis was performed using GraphPad Prism version 8.0 (GraphPad Software Inc., San Diego, CA, USA). One-way ANOVA and Student’s *t*-test were used. Results are given as means with the standard deviation. Results were considered statistically significant when (*) *p* ≤ 0.05, (**) *p* ≤ 0.01, (***) *p* ≤ 0.001, and (****) *p* ≤ 0.0001.

## 3 Results and Discussion

### 3.1 Synthesis of Radioactive ^198^AuNPs

Radioactive gold nanoparticles ^198^AuNPs were synthesized in the same way as nonradioactive nanoparticles.^13^ The preparation was carried out with the use of ^198^Au suspended in water. The method of dissolving the target is described in detail in subsection 2.2. The size (30 nm), zeta potential, and shape were confirmed using high resolution transmission electron microscopy (HR-TEM) and dynamic light scattering (DLS) techniques. All physicochemical properties of ^198^AuNPs were determined based on “cold” nanoparticles.^13^

### 3.2 Synthesis and Characterization of DOX- ^198^AuNPs- Tmab

**Scheme 1:**
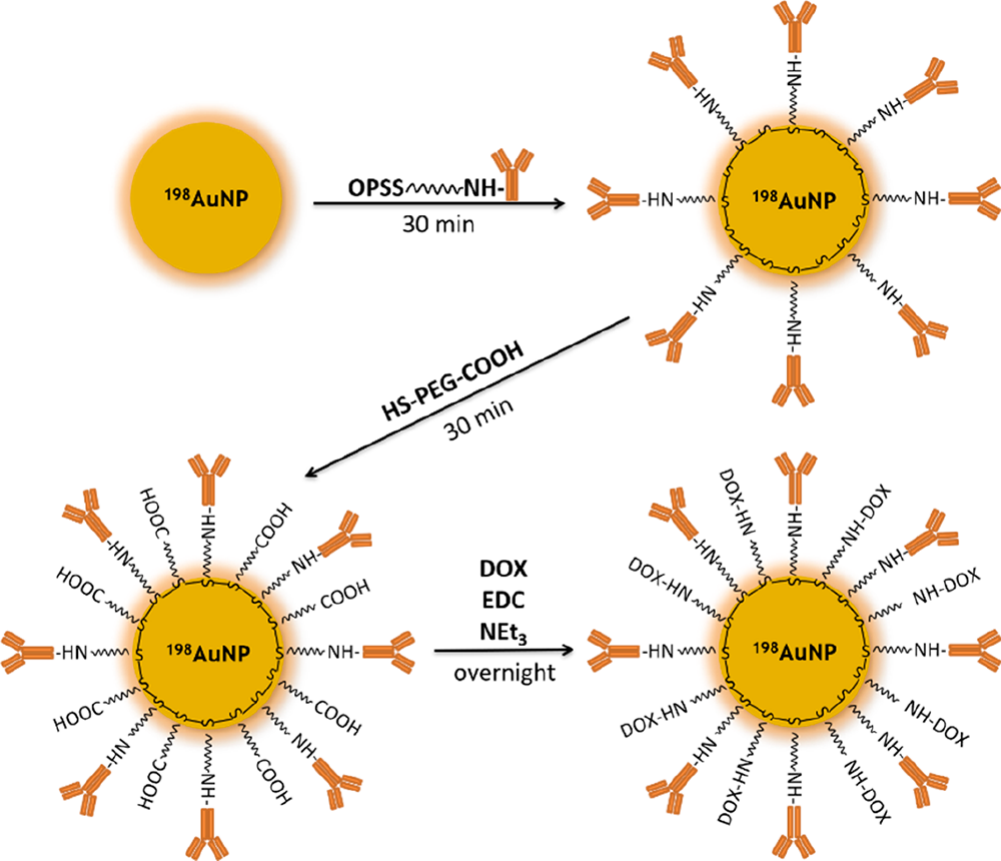
Synthesis of DOX-^198^AuNPs-Tmab Radiobioconjugate

Functionalization of the ^198^AuNPs’ surface with PEG-Tmab, further PEGylation, and finally DOX conjugation were performed in accordance to our previously published methods.^13^ A schematic representation of the synthesis of the radiobioconjugate is shown in Scheme 1. According to our calculations, 72.6 ± 7.9 Tmab molecules were attached to one radioactive nanoparticle, which was afterward linked to DOX.^13^

### 3.3 Binding and Internalization Studies

**Figure 1:**
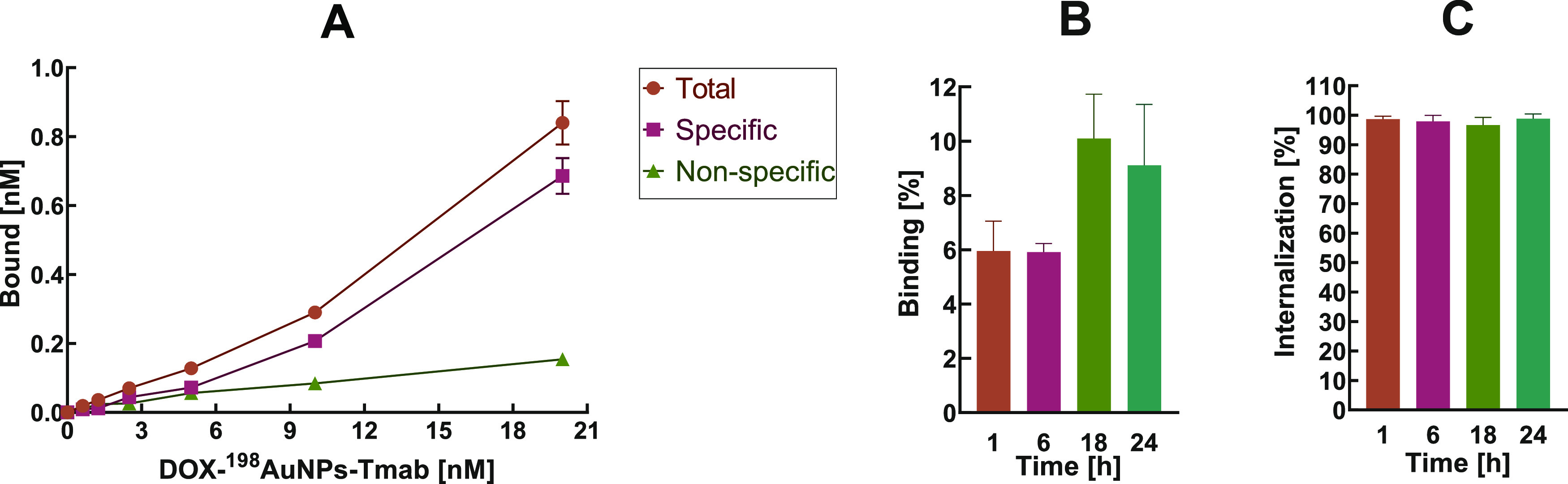
Binding and Internalization Studies of DOX-^198^AuNPs-Tmab. Specificity of DOX-^198^AuNPs-Tmab binding on SKOV-3 (HER2+) cells; (B) percentage of radiobioconjugate binding in SKOV-3 (HER2+) cells; and (C) percentage of radiobioconjugate internalization in SKOV-3 (HER2+) cells.

Binding and internalization experiments were carried out to investigate the targeting efficiency of the synthesized radiobioconjugate DOX-^198^AuNPs-Tmab. The study was performed on SKOV-3 (HER2+) and MDA-MB-231 (HER2-) cell lines. As shown in Figure 1A, the DOX-^198^AuNPs-Tmab radiobioconjugate exhibited high affinity to HER2 receptors overexpressed in SKOV-3 cells. The maximum percentage of binding was achieved after 18 h of incubation with the radiobioconjugate (10.1 ± 1.6%; Figure 1B). In the case of HER2- MDA-MB-231 cells, no receptor affinity was observed. Comparable results confirming the specific binding of gold nanoparticles conjugated with trastuzumab have been reported previously.^13−15^ Internalization experiments revealed a high and rapid uptake of the nanoconjugate into the cells. After one hour, 98.7 ± 1.0% of the specifically bound radiobioconjugate had already been internalized (Figure 1C). At subsequent time points (6, 18, and 24 h) the results remained similar, and are in agreement with other studies.^16,17^

### 3.4 Cytotoxicity Studies

#### 3.4.1 MTS Assay

**Figure 2:**
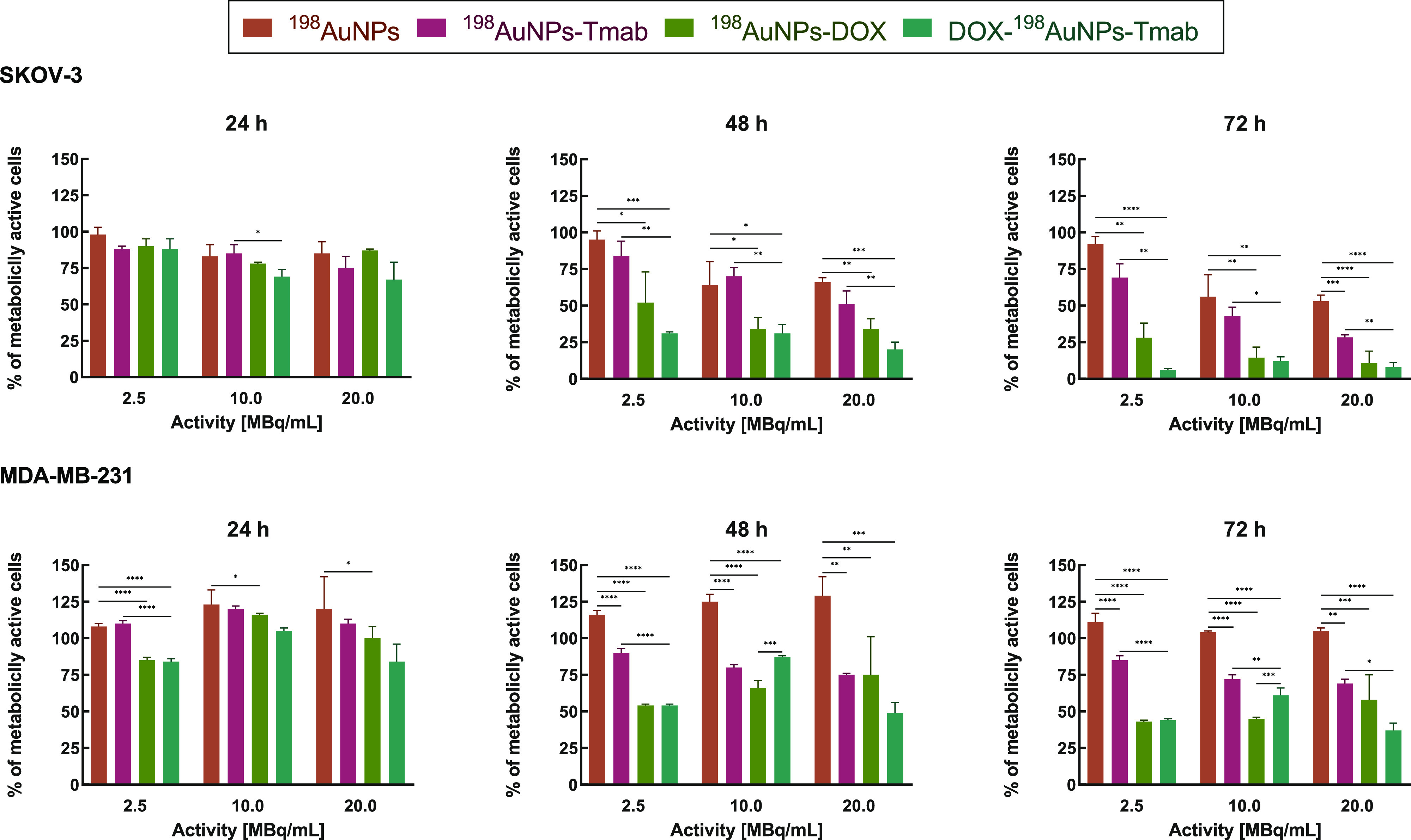
Summary of metabolic activity of SKOV-3 (HER2+) and MDA-MB-231 (HER2-) cells treated with ^198^AuNPs, ^198^AuNPs-Tmab, ^198^AuNPs-DOX, and DOX-^198^AuNPs-Tmab radioconjugates at three activity doses (2.5, 10, and 20 MBq/mL) after 24, 48, and 72 h. Doxorubicin concentration was 7 μg/mL. Untreated cells were used as control. Data represent the mean ± SD, *n* = 3.

The cytotoxic effect of ^198^AuNPs, ^198^AuNPs-Tmab, ^198^AuNPs-DOX, and DOX-^198^AuNPs-Tmab was tested on SKOV-3 and MDA-MB-231 cells, which were treated with three different doses of compounds (2.5, 10, and 20 MBq/mL). The absorbance was measured at three time points (24, 48, and 96 h) after cell treatment. The obtained results are presented in Figure 2. As expected, similarly to our previous work,^13^ a weaker cytotoxic effect of the compounds was observed on the MDA-MB-231 cells, due to the lack of HER2 receptors. For all activities tested, the viability decreased in a dose- and time-dependent manner. As shown in Figure 2, ^198^AuNPs at the lowest dose (2.5 MBq/mL) caused a negligible degree of cytotoxicity on SKOV-3 cells, with a 92.0 ± 5.2% survival rate after 72 h. At higher doses, viability decreased (56 ± 15% for the 10 MBq/mL dose and 53.0 ± 4.2% for the 20 MBq/mL dose at 72 h). Radioactive gold nanoparticles with the attached Tmab vector showed a stronger toxic effect in comparison to the nonfunctionalized nanoparticles. After 72 h, the percentage of metabolically active cells was 69.2 ± 9.4%, 42.8 ± 6.1%, and 28.3 ± 1.7%, from the lowest to the highest dose, respectively. However, the radioactive gold nanoparticles with the attached chemotherapeutic agent (^198^AuNPs-DOX) were more cytotoxic, as after 3 days of incubation only 28 ± 10% of cells were determined as viable even at the lowest dose. When higher doses were used, greater toxicity was observed (85.6 ± 7.3% for the 10 MBq/mL dose; 89.2 ± 8.1% for the 20 MBq/mL dose). It is worth noting that the most potent compound appeared to be the DOX-^198^AuNPs-Tmab radiobioconjugate.

The greatest differences in the evaluation of the cytotoxic effect of the DOX-^198^AuNPs-Tmab compared to the other compounds were observed at the lowest dose (2.5 MBq/mL) at 48 and 72 h. Cytotoxicity evaluation at 72 h revealed an additive interaction between ^198^AuNPs-Tmab and ^198^AuNPs-DOX. Additionally, the results after 48 h showed that the combination of the chemotherapeutic agent and radiation was more effective (15% higher effect) than when each agent was used alone. In this experiment, we obtained a greater cytotoxic effect than in similar studies, where nanoparticles were tested with doxorubicin.^13,18−20^ Moreover, comparable results have been reported with ^177^Lu-NP-mAb (mAb - trastuzumab), where the conjugation of the monoclonal antibody to ^177^Lu-NPs was shown to induce stronger cytotoxicity in relation to ^177^LuCl_3_ and ^177^Lu-NPs.^21^

#### 3.4.2 Apoptosis Assay

**Figure 3:**
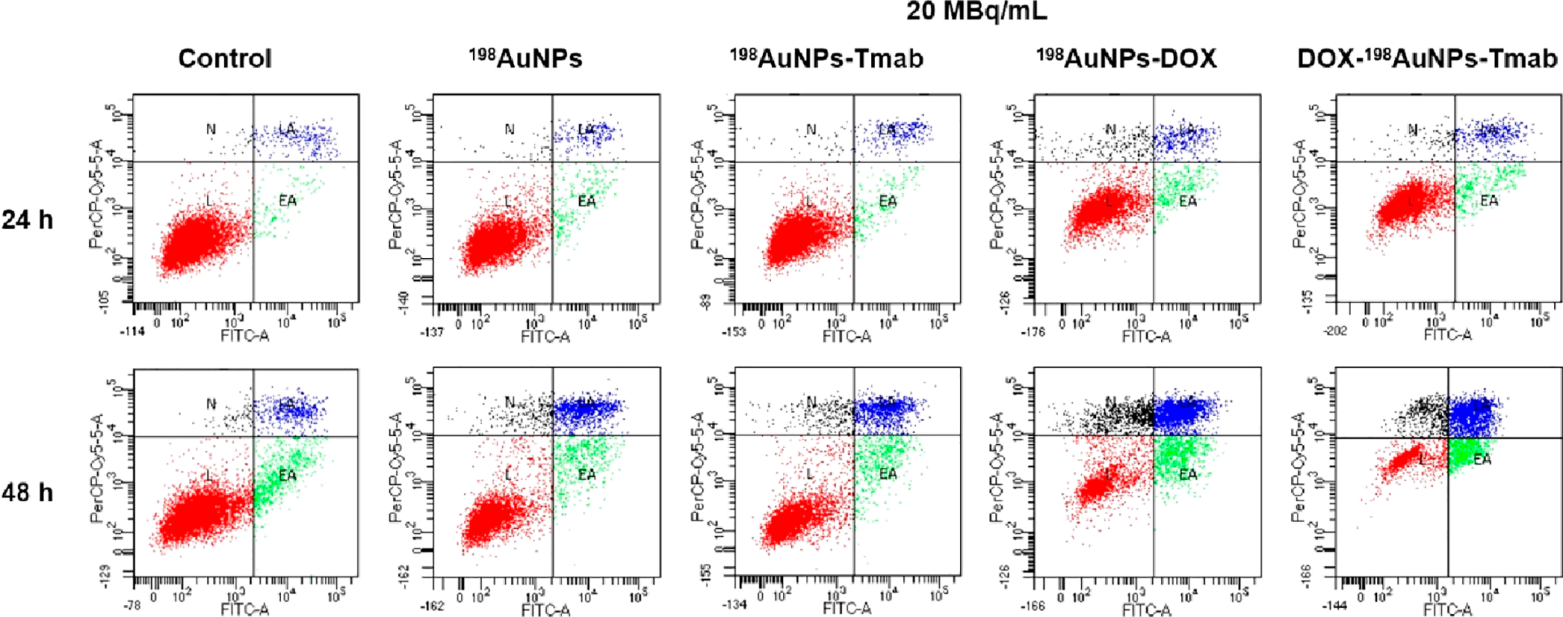
Effect of ^198^AuNPs, ^198^AuNPs-Tmab, ^198^AuNPs-DOX and DOX-^198^AuNPs-Tmab on cell apoptosis. Representative graphs of the flow cytometry analysis after the treatment with 20 MBq/mL of ^198^AuNPs, ^198^AuNPs-Tmab, ^198^AuNPs-DOX and DOX- ^198^AuNPs-Tmab and with no treatment (control) for 24 and 48 h. L - live cells (red), EA - early apoptosis (green), LA - late apoptosis (blue), N - necrosis (black).

For an in-depth evaluation of the effectiveness of the radiobioconjugate, an apoptosis assay was performed using flow cytometry (Figure 3).

**Figure 4:**
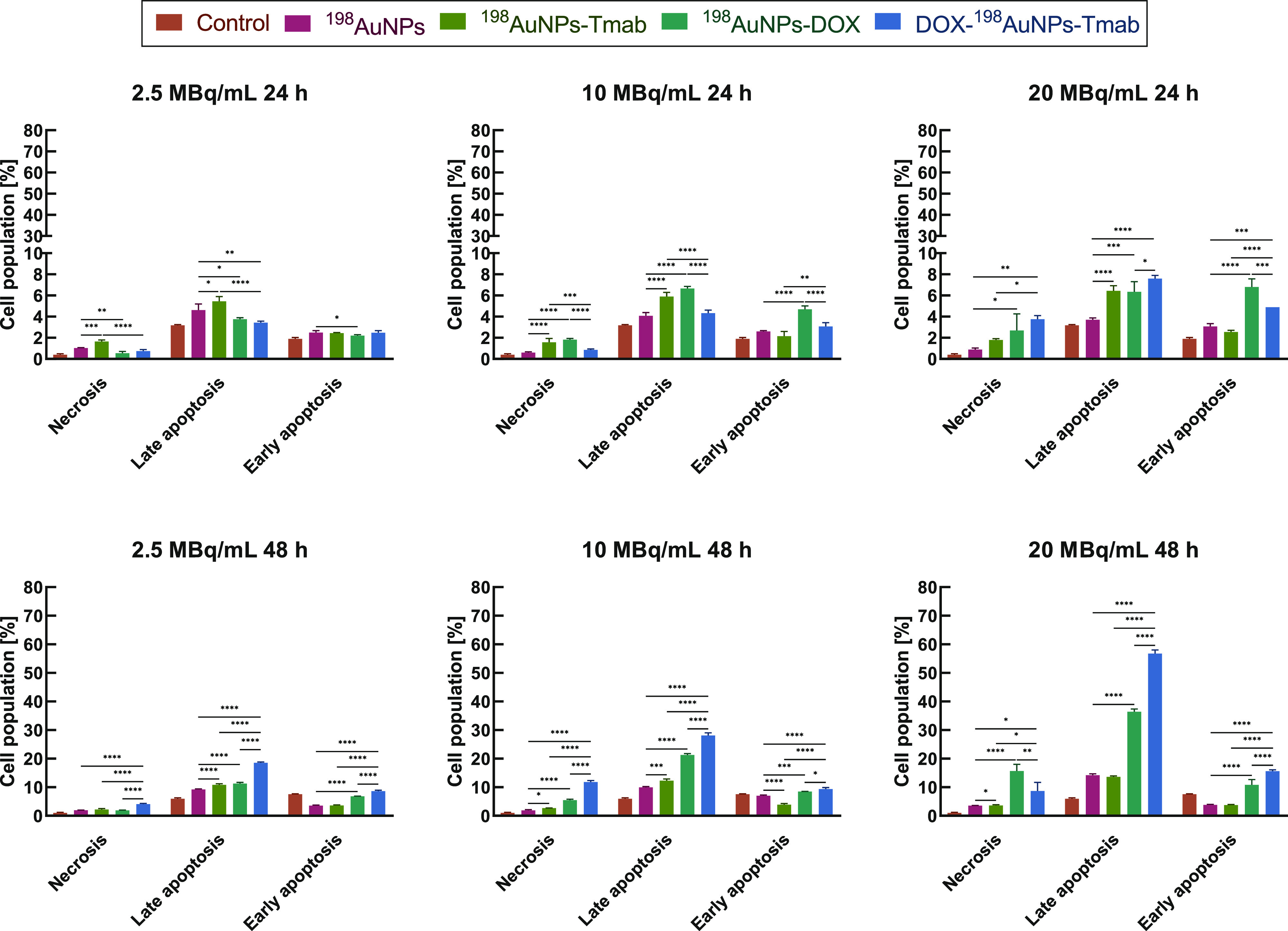
Apoptosis assay: distribution of cell populations (necrosis, late and early apoptosis) treated with the following compounds: ^198^AuNPs, ^198^AuNPs-Tmab, ^198^AuNPs-DOX (7 μg/mL of DOX) and DOX-^198^AuNPs-Tmab (7 μg/mL of DOX) after 24 and 48 h. Untreated cells were used as the control. Results are the mean ± SD, *n* = 4.

The obtained results are listed in Figure 4. On the first day after the treatment, fractions of necrotic and early apoptotic cells slightly increased. After 48 h, a significant increase in apoptotic cells, compared to untreated cells and to those treated with control compounds (^198^AuNPs-Tmab, ^198^AuNPs-DOX) was observed. Most cells died by apoptosis. The presence of cells in an early stage of apoptosis was observed after 24 hours, and decreased with time, followed by an increased presence of cells in the late stage of apoptosis. After 48 h, 18.55 ± 0.29% of cells were detected as late apoptotic for the 2.5 MBq/mL dose of radiobioconjugate, whereas 4.08 ± 0.27% were early apoptotic. The higher dose of 10 MBq/mL led to greater apoptosis, i.e. 28.08 ± 0.93% (late apoptosis) and 11.83 ± 0.51% (early apoptosis), respectively. Most importantly, the dose of 20 MBq/mL of DOX-^198^AuNPs-Tmab was the most efficient in triggering apoptosis, i.e. 56.8 ± 1.3% of the cells died by late apoptosis, while 15.60 ± 0.50% died by early apoptosis. In comparison, the ^198^AuNPs-DOX compound was not as effective as DOX-^198^AuNPs-Tmab (36.43 ± 0.95% late apoptosis and 10.8 ± 1.9% early apoptosis). Radiobioconjugate effects showed a definitive increase in the number of late apoptotic cells, which is consistent with the prediction and available knowledge that radiation causes activation of apoptotic pathways in tumor cells.^22^ Additionally, the conjugation of DOX also has great potential in inducing apoptosis as has been commonly mentioned in literature, including our previous work.^13^

#### 3.4.3 Spheroids

**Figure 5:**
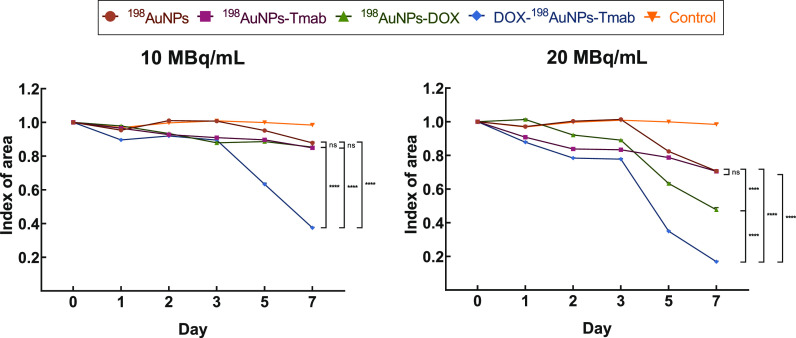
Time-dependent surface development characteristics of control SKOV-3 spheroids after treatment with ^198^AuNPs, ^198^AuNPs-Tmab, ^198^AuNPs-DOX and DOX-^198^AuNPs-Tmab. Untreated cells were used as the control. Data represent the mean ± SD, *n* = 3.

**Figure 6:**
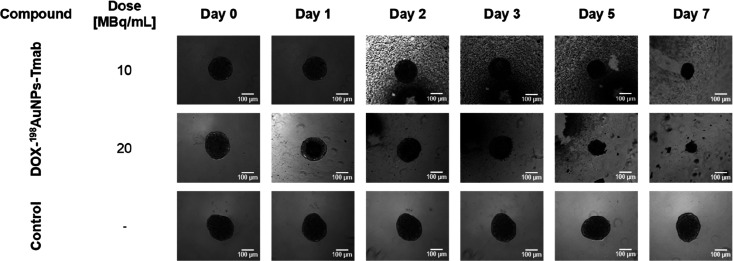
Microscope images of the measured control and DOX-^198^AuNPs-Tmab-treated SKOV-3 spheroids (10 and 20 MBq/mL).

Based on literature data that 3D cell colonies more accurately represent tumor models than 2D cell cultures,^23,24^ a cytotoxicity study on spheroids was performed (see Figures 5 and 6). Microscope images showed that all tested compounds inhibited spheroid growth (Figure 6). The study was terminated when the spheroid treated with the highest dose of radiobioconjugate (DOX-^198^AuNPs-Tmab) decreased by almost 6-fold (day 0: 139 262 ± 328 μm^2^ vs day 7: 23 406 ± 519 μm^2^). At the same period of time (7 days), treatment with 20 MBq/mL of ^198^AuNPs-DOX halved the spheroid area (day 0: 131 426 ± 376 μm^2^ vs day 7: 62 603 ± 1717 μm^2^), while the ^198^AuNPs-Tmab caused reduction of the area by ∼30% (day 0: 139 222 ± 151 μm^2^ vs day 7: 98 219 ± 525 μm^2^). As expected, the strongest therapeutic effect was obtained for the highest dose of the multimodal radiobioconjugate. The 3D model studies confirmed the enhanced cytotoxic effect of DOX-^198^AuNPs-Tmab and showed great promise for proceeding to in vivo studies.

### 3.5 Cell Cycle Assay

**Figure 7:**
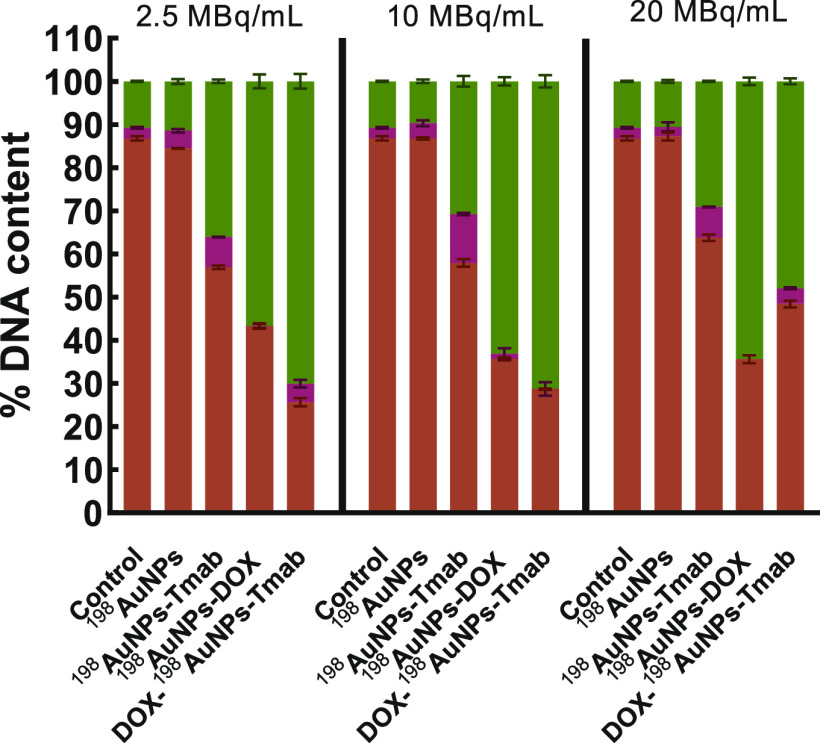
Cell cycle distribution of cell phases (G_0_/G_1_- brown color, S – pink color and G_2_/M – green color) treated with the following compounds: ^198^AuNPs, ^198^AuNPs-Tmab, ^198^AuNPs-DOX (7 μg/mL of DOX) and DOX-^198^AuNPs-Tmab (7 μg/mL of DOX) after 24 h. Untreated cells were used as control. Results are the mean ± SD, *n* = 4.

To investigate the effect of DOX-^198^AuNPs-Tmab on HER2+ cells, a cell cycle study using a flow cytometry method was performed (Figure 7). From the data obtained after treatment with all compounds, cells showed increased arrest in the G_2_/M cellular phase. An enhancement in the S-phase was observed in nanoparticles with attached trastuzumab, as confirmed by a study performed by Mayfield et al., where the cell cycle after Herceptin treatment in breast cancer cell lines was investigated.^25^ Moreover, doxorubicin is also reported to induce cell cycle arrest in G_2_/M and S phases.^26,27^ However, in this study the proportion of the G_2_/M phase significantly increased when the cells were treated with ^198^AuNPs-DOX. In the case of the DOX-^198^AuNPs-Tmab radiobioconjugate, an increase in the S-phase is due to the effect of trastuzumab,^25^ but the growth in the G_2_/M phase is probably caused by the radiation^28^ as well as the presence of DOX.^29^

### 3.6 Ex Vivo Biodistribution Studies

**Figure 8:**
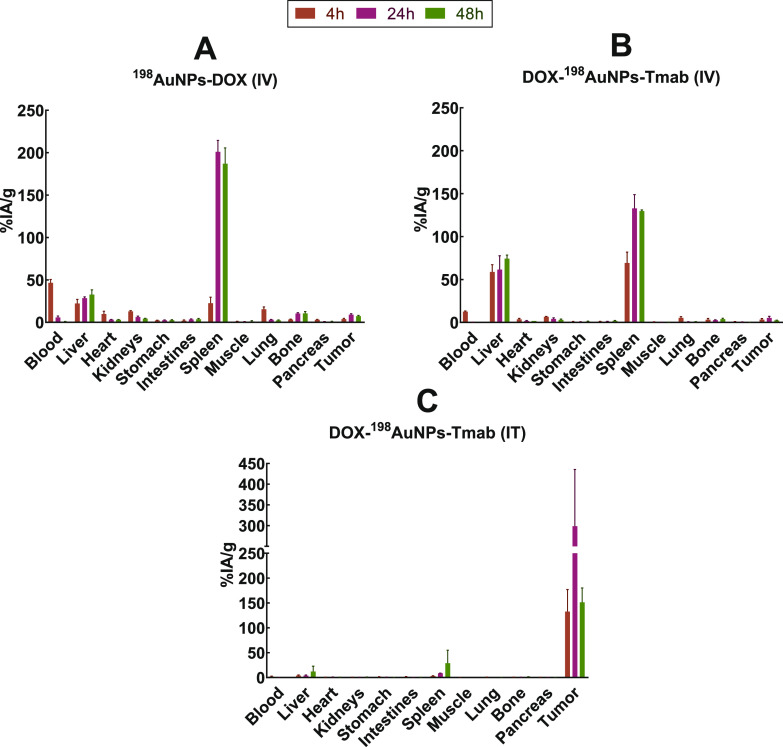
Ex vivo biodistribution after 4, 24, and 48 h of (A) ^198^AuNPs-DOX (intravenous injection; average dose 5.75 ± 0.23 MBq), (B) DOX-^198^AuNPs-Tmab (intravenous injection; average dose 6.16 ± 0.20 MBq), and (C) DOX-^198^AuNPs-Tmab (intratumoral injection; average dose 6.30 ± 0.18 MBq) in mice bearing SKOV-3 tumors (*n* = 3, mean ± SD). Uptake in each organ was expressed as a percentage of injected activity per gram (% IA/g).

Ex vivo biodistribution data of ^198^AuNPs-DOX and DOX-^198^AuNPs-Tmab in SCID mice with SKOV-3 tumors are depicted in Figure 8. More specifically, Figure 8A and 8B present the results after intravenous administration of the aforementioned agents. The highest accumulation was noted in the organs of the reticuloendothelial system (RES) such as the liver and spleen, where 22.2 ± 4.8% and 22.5 ± 7.1% IA/g (respectively at 4 h p.i.), 28.5 ± 1.5% and 201.0 ± 14% IA/g (respectively at 24 p.i.), and 32.7 ± 5.5% and 187 ± 19% IA/g (respectively at 48 p.i.) were observed for ^198^AuNPs-DOX (Figure 8A). There was also little increase in the accumulation of the compound in the bloodstream at the first time point (4 h), while the lack of measured activity at subsequent points is indicative of clearance. Similar observations were noted with the DOX-^198^AuNPs-Tmab compound (Figure 8B), where accumulation in the liver and spleen was 58.8 ± 8.4% and 69 ± 13% IA/g (respectively at 4 h p.i.), 62 ± 16% IA/g, 133 ± 16% IA/g (respectively at 24 p.i.), and 74.3 ± 4.2% and 129.7 ± 1.4% IA/g (respectively at 48 p.i.). Our results are consistent with several investigations showing that the intravenously injected NPs are predominantly retained in the liver and spleen.^30−33^ Rapid blood clearance and negligible tumor uptake for ^198^AuNPs-DOX and DOX-^198^AuNPs-Tmab at both time points (24 and 48 h p.i.) were observed. The increased uptake of DOX-^198^AuNPs-Tmab in the RES organs in comparison to ^198^AuNPs-DOX is due to the larger size of the radiobioconjugate bearing trastuzumab.^13^ Hirn et al. found that nanoparticle uptake in the liver was size-dependent, after performing the biodistribution of surface-modified monosulfonated triphenylphosphine (TPPMS) ^198^AuNPs.^34,35^ Furthermore, there are other reports indicating that after intravenous administration, proteins strongly affect nanoparticle biodistribution, causing their accumulation in the liver.^36^ In other studies, intravenously administered 20 and 40 nm AuNPs were rapidly accumulated in the spleen and liver, with a high percentage remaining at these organs for up to six months post-administration.^37^ Based on literature data, spherical nanoparticles in the range of 20–150 nm are mostly entrapped within the liver and spleen.^38^ This may be explained by the fact that fenestrated, discontinuous endothelia allow nanoparticles (up to 100 nm) to transfer from the bloodstream into the parenchyma. Moreover, nanoparticle accumulation in the organs of the reticuloendothelial system can occur via opsonization, which means that nanoparticles could bind to antibodies in the plasma and then get recognized by the phagocyte-rich RES.^37,39^

Apart from intravenous injection, the DOX-^198^AuNPs-Tmab radiobioconjugate was also directly administered to the tumor (Figure 8C, intratumoral injection of DOX-^198^AuNPs-Tmab). As expected, almost all the radiotracer remained at the injection site, resulting in high tumor uptake (% IA/g: 91% at 4 h p.i., 95% at 24 h p.i. and 77% at 48 h p.i.). Other organs such as the liver and spleen demonstrated lower uptake, which was less than 21% of the total distributed activity %IA/g. The low accumulation in the organs of the reticuloendothelial system suggests minor leakage of the compound from the tumor.

### 3.7 Therapeutic Efficacy Studies

**Figure 9:**
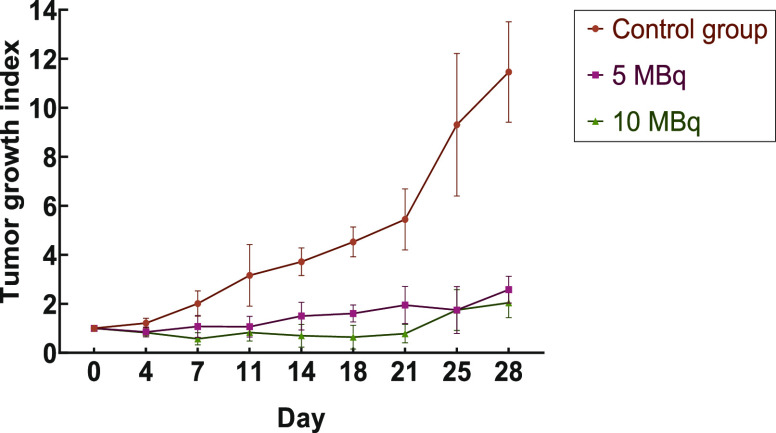
Therapeutic efficacy study of two doses of DOX-^198^AuNPs-Tmab (dose A ≈ 5 MBq: 5.16 ± 0.10 MBq; dose B ≈ 10 MBq: 9.99 ± 0.22 MBq) after single-dose intratumoral injection in mice bearing SKOV-3 tumors (*n* = 3, mean ± SD). Animals in the control group were treated with saline. The study was conducted for up to 4 weeks.

**Figure 10:**
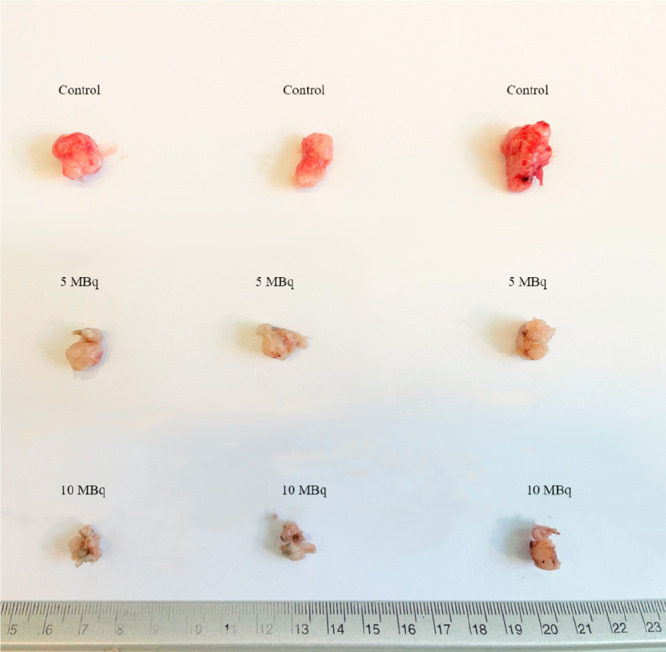
Representative images of excised tumors at the end of the therapeutic efficacy study, 28 days p.i. of DOX-^198^AuNPs-Tmab: Upper panel: control group of mice injected with 0.9% NaCl; Middle panel: therapy group of mice injected with ∼5 MBq DOX-^198^AuNPs-Tmab; Lower panel: therapy group of mice injected with ∼10 MBq DOX-^198^AuNPs-Tmab.

Therapeutic efficacy was determined by estimating the tumor growth index (TGI) of SKOV-3 xenografts treated with a single injection of DOX-^198^AuNPs-Tmab at two doses (Dose A, 5.16 ± 0.10 MBq; Dose B, 9.99 ± 0.22 MBq). After 4 weeks of evaluating tumor progression and observation of the overall condition of the mice, the study was terminated. At the end of the study, mean tumor volume of the control group was 909 ± 167 mm^3^, 503 ± 113 mm^3^ in Group A and 299 ± 47 mm^3^ in Group B. As presented in Figure 9, Dose A reduced tumor size by 77.5 ± 8.8%, while an 82.2 ± 8.5% tumor volume reduction was observed for Dose B, in comparison to the tumor volume of the mice in the saline treated group. The effect of the dose on tumor shrinkage is also observed in Figure 10. Our results are comparable with those of other studies, in which radioactive AuNPs have been used for anticancer therapy. In the work of Chanda et al., gum arabic glycoprotein (GA)-functionalized AuNPs with a hydrodynamic diameter of 85 nm (similar to DOX^–^AuNPs-Tmab size: 79.9 ± 4.4 nm^13^) were intratumorally administered to prostate tumor-bearing SCID mice.^30^ It was observed that 21 days post-injection (p.i.) of a single GA-^198^AuNPs dose of 408 μCi (15.1 MBq, 70 Gy), tumor volume was 82% smaller when compared to the tumor volume of the control group. In another study, Shukla et al. proposed epigallocatechin-gallate (EGCg) functionalized radioactive gold nanoparticles as therapeutic agents. The performed experiments demonstrated a 5-fold reduction in prostate tumors relative to the control (untreated) group 28 days after injection of 5.032 MBq (136 μCi).^31^ Comparative results of intratumorally administered mangiferin-functionalized radioactive gold nanoparticles MGF–^198^AuNPs (5.92 MBq, 160 μCi) have also been reported.^32^

## 4 Conclusions

We have shown that radioactive gold nanoparticles modified with DOX and trastuzumab exhibit great potential for targeted therapy of HER2+ cancers. The synthesized DOX-^198^AuNPs-Tmab radiobioconjugate was shown to have high receptor affinity as well as cytotoxicity toward ovarian cancer cells expressing HER2 receptors. Our therapeutic efficacy studies on mice demonstrated an 82% reduction of tumor growth after a single-dose (10 MBq) intratumoral injection of DOX-^198^AuNPs-Tmab. Significant uptake in nontargeted organs like spleen and liver limits the use of this compound in standard intravenous treatments. This radiopharmaceutical could be applied as a nanobrachytherapy agent by intratumoral or post-resection injection. Nevertheless, based on these results, local therapy with the use of the developed multimodal radiobioconjugate seems to be very promising, due to the strong synergistic effect attributed to the simultaneous presence of Tmab, DOX, and the β emitter - ^198^Au.

In conclusion, a low concentration of DOX combined with β radiation could potentiate the antitumor effect of the drug on HER2+ cancer cells, thus overcoming the side effects caused by conventional chemotherapy with the aforementioned drug.
